# Not a Disease of the Past: A Case Report of Heroin-Induced Toxic Leukoencephalopathy Causing Progressive Neurologic Decline

**DOI:** 10.7759/cureus.103350

**Published:** 2026-02-10

**Authors:** M.Nour Chabalout, Mohamed Darwish, Imad Alabdul Razzak, Mohanad Alkhalaila

**Affiliations:** 1 Internal Medicine, Tower Health Medical Group, Phoenixville, USA

**Keywords:** cerebellar abnormalities, gait ataxia, heroin, heroin-induced leukoencephalopathy, neuroimaging findings, substance abuse side effects

## Abstract

Toxic leukoencephalopathy is a rare neurological complication associated with various toxins, including inhaled heroin (“chasing the dragon”). We report a case of a 36-year-old female with a history of heroin dependence, in remission for seven years until a recent relapse, who presented with progressive deterioration in speech clarity, swallowing difficulty, and imbalance. Neuroimaging revealed diffuse leukoencephalopathy involving the cerebellar white matter and internal capsules, suggestive of heroin induced leukoencephalopathy. Laboratory evaluation was unremarkable, and infectious, autoimmune, and metabolic causes were considered less likely. This case highlights the importance of recognizing heroin-induced toxic leukoencephalopathy as a cause of subacute progressive neurological decline in patients with recent heroin use, even in the absence of intravenous administration.

## Introduction

Heroin-induced toxic leukoencephalopathy (HLE) is a rare but severe neurological complication most strongly associated with the "chasing the dragon" method of heroin inhalation, in which heroin is heated on aluminium foil, and the resulting vapours are inhaled. This practice leads to the formation of pyrolysate, which is believed to contain neurotoxic compounds that damage cerebral white matter. Opioid addiction is a serious and widespread public health problem, affecting approximately 2.4 million Americans, nearly one million of whom report heroin use. The economic burden of heroin abuse in the United States is substantial, exceeding $51 billion annually. Inhaled heroin use has emerged as a global concern and is rapidly reaching epidemic proportions, particularly among urban youth [1].

Based on the most recent comprehensive data available, approximately 141 cases of heroin-induced leukoencephalopathy had been reported through 2018, with the majority (82.3%, or 116 cases) associated with "chasing the dragon" (CTD) and the remaining 17.7% (25 cases) from other inhalation methods. Clinically, affected individuals typically present with a spectrum of neurobehavioral and motor symptoms. Early signs may include inattentiveness, confusion, ataxia, and psychomotor slowing [1]. As the condition progresses, patients can develop corticospinal or extrapyramidal signs, severe confusion, delirium, and, in the most severe cases, generalized motor impairment, abulia, and coma, often with a high risk of mortality. MRI findings are characteristic, showing diffuse, symmetric white matter hyperintensities, especially in the cerebellum, posterior cerebrum, internal capsules, and brainstem, with spongiform degeneration on pathology [1-5].

The pathophysiology is not fully understood, but evidence points to mitochondrial dysfunction and impaired energy metabolism in oligodendrocytes, leading to intra-myelinic vacuolation and spongiform changes. While aluminium exposure from foil was once suspected, epidemiological and pathological data do not support a direct causal role for aluminium [1]. The syndrome is distinct from leukoencephalopathy seen with other routes of heroin use, both in clinical presentation and neuroimaging patterns [3]. Prognosis is variable: some patients recover fully or partially over months to years, but many others experience persistent deficits or death, highlighting the importance of early recognition and supportive care, which mainly consists of folate, thiamine, physical therapy, and coenzyme Q10 [6].

## Case presentation

A 36-year-old female with a history of heroin dependence (in remission for seven years), not known to have other past medical history, presented with progressively worsening slurred speech, generalized weakness, gait imbalance, and difficulty swallowing liquids for one week. She reported unintentional weight loss and intermittent double vision. Patient denied fever, cough, chest pain, abdominal pain, or recent intravenous drug use. She relapsed into inhalational heroin use earlier in the three months prior to admission; however, she stopped using heroin three weeks prior to admission and completed inpatient detoxification 10 days prior to the onset of her symptoms. She denied any drug use since discharge.

On examination, she was alert and oriented with intact cognition. Neurological examination revealed dysarthria, bilateral upper extremity weakness (4/5 handgrip), bilateral lower extremity weakness (3/5 iliopsoas), truncal ataxia, limb ataxia (left greater than right), wide-based gait, and mild head tremor. Reflexes were symmetric without upper motor neuron signs. Cranial nerves were intact. There were no sensory deficits, and the remainder of the physical examination was unremarkable.

Laboratory evaluation, including complete blood count, metabolic panel, and inflammatory markers, was within normal limits (Table 1). A urine drug screen was also negative for illicit substances. Vitamin D deficiency was noted and considered common in patients with opioid use disorder. Aspartate aminotransferase was mildly elevated, with plans for repeat testing in four weeks.

**Table 1:** Key Laboratory Findings.

Test	Result	Normal Range
White Blood Cell (×10³/µL)	8	4.0-10.5
Hemoglobin (g/dL)	14	12.0-16.0
Platelets (×10³/µL)	300	150-400
Aspartate Aminotransferase (AST, U/L)	54	10-40
Alanine Aminotransferase (ALT, U/L)	47	7-56
C-Reactive Protein (mg/dL)	<0.5	<1.0
Thyroid-Stimulating Hormone (µIU/mL)	0.95	0.4-4.5
Folic Acid (ng/mL)	8.8	3.0-17.0
Vitamin B12 (pg/mL)	321	200-900
Vitamin D, Total (ng/mL)	30	30-100
Lyme Disease Antibodies (IgG, IgM)	Negative	Negative
HIV Antibodies 1+2	Negative	Negative

**Figure 1:**
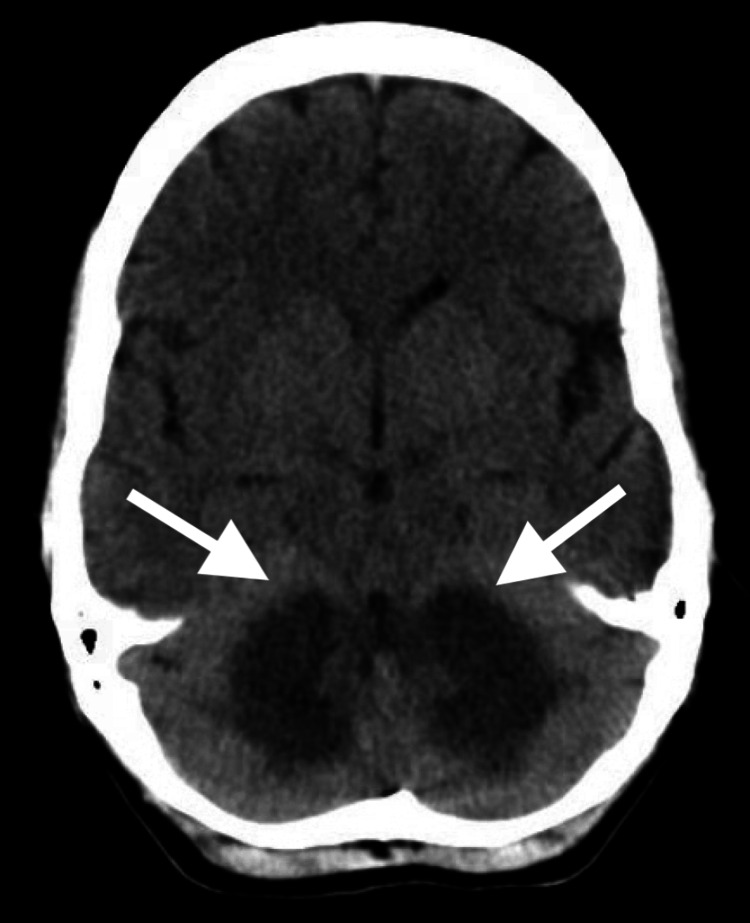
Noncontrast CT scan of the brain demonstrating diffuse hypoattenuation in the cerebellar white matter (arrows).

An initial CT scan of the brain showed diffuse hypoattenuation in the cerebellar white matter and bilateral internal capsules (Figure 1).

**Figure 2:**
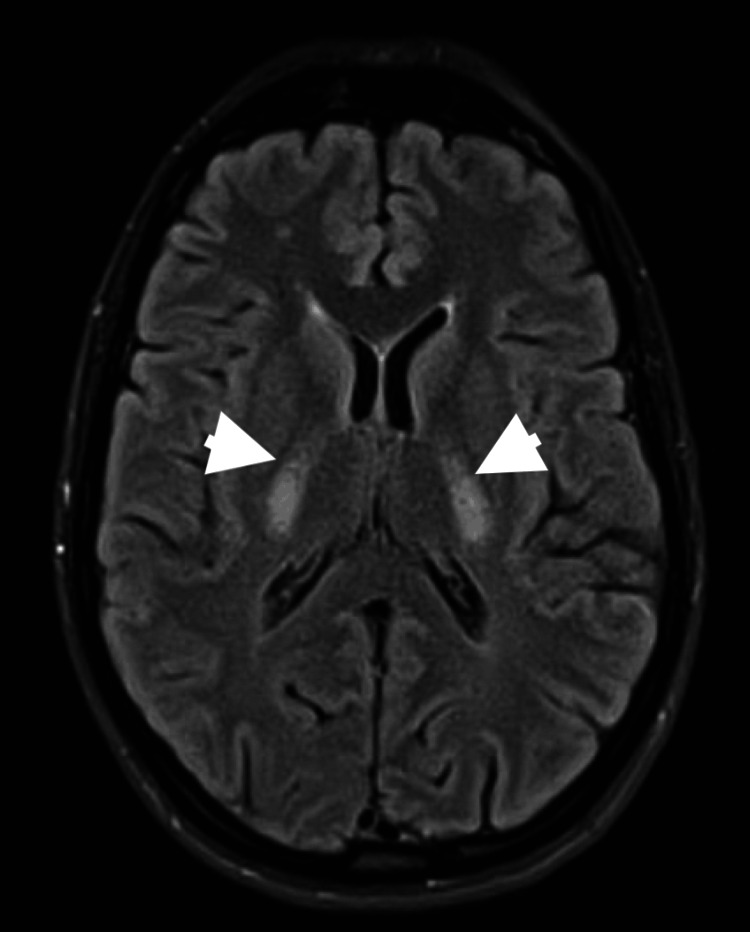
Axial MRI fluid-attenuated inversion recovery (FLAIR) sequence demonstrating symmetric hyperintensities in the bilateral internal capsules (arrowheads).

**Figure 3:**
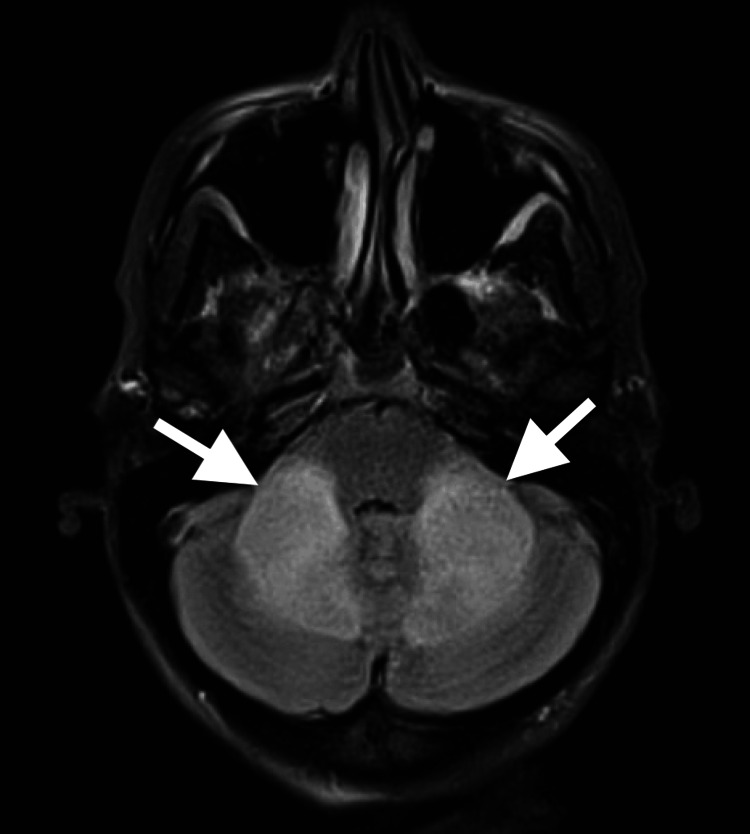
Axial MRI fluid-attenuated inversion recovery (FLAIR) sequence demonstrating hyperintensities in the middle cerebellar peduncles (arrows).

MRI of the brain was done and confirmed diffuse symmetrical white matter hyperintensity involving the cerebellar white matter, middle cerebellar peduncles, and posterior limbs of the internal capsules (Figures 2, 3).

The differential diagnosis included heroin-induced leukoencephalopathy, other toxic leukoencephalopathies, and demyelinating disease. Infectious etiologies were considered less likely given the absence of infectious signs, symptoms, and negative blood workup, including negative blood and urine cultures. Neurology consultation was obtained, and a lumbar puncture (LP) was planned, but the patient declined it. Demyelinating diseases, such as multiple sclerosis, were ruled out because of symptom onset and characteristic findings on MRI of the brain; however, LP was not done eventually, as the patient did not consent to it.

Supportive therapy with thiamine, folate, B-complex vitamins, vitamin C, coenzyme Q10, and vitamin B12 was initiated. A psychiatry consult was then obtained. Extensive counselling on heroin use was done, and the patient showed great interest in quitting and joining a rehabilitation program. Physical therapy was eventually started as part of the whole management plan, with the patient showing gradual improvement in her gait and balance on a daily basis. Subsequently, she was discharged to a rehabilitation center to continue treatment.

## Discussion

HLE typically presents with subacute progression of cerebellar and pyramidal signs, including dysarthria, ataxia, and weakness, as seen in our patient. More severe cases have been reported to mimic “locked-in syndrome,” in which patients are unable to move or speak despite being awake and attentive-a condition known as akinetic mutism-occurring after prolonged heroin inhalation leading to extensive white matter damage [7,8].

The risk of developing toxic leukoencephalopathy correlates with the duration and intensity of heroin exposure, as longer-term use is linked to more extensive and severe white matter damage. Early recognition and cessation of heroin use are essential for management. Patients with mild leukoencephalopathy who stop heroin use early typically survive and retain independence in daily activities. At the same time, those with moderate or severe disease face a guarded prognosis and a higher risk of death or long-term disability [1]. Heroin can be inhaled by the “chasing the dragon” technique or by smoking tobacco laced with heroin. In a 2019 study analyzing 50 cases of HLE reported between 1994 and 2018, inhalation was identified as the most common route (60%), followed by intravenous injection (30%) and insufflation (10%) [3]. This finding suggests that the theory attributing HLE solely to combustion by-products is incomplete, as it fails to account for the 40% of cases related to non-inhalational use.

Although data on the pharmacokinetics of inhaled heroin are limited, absorption via inhalation is known to be extremely rapid due to the lipophilic nature of the drug [9]. We propose that the white matter inflammation and damage seen in HLE may result from the transiently high concentrations of heroin in the brain, which are directly proportional to its rapid absorption rate. The systemic bioavailability of heroin likely plays a lesser role in HLE pathophysiology, as previously published studies measured heroin concentrations in peripheral blood, which do not accurately reflect transient peak levels within the central nervous system.

There is no established cure for HLE yet. Treatment is mainly supportive, focusing on intensive care, rehabilitation, physical therapy, and nutritional support [10]. Coenzyme Q10, which is an antioxidant, has been mentioned in some case reports and case series. Some studies have shown neurological recovery in patients receiving antioxidants, suggesting a possible benefit, though this is not yet a standard of treatment [2,3,10].

## Conclusions

Heroin use remains a major public health challenge with devastating neurological and systemic consequences, as illustrated by this case. We aim to increase the awareness of HLE among healthcare providers to facilitate early recognition, which can play an important role in the management of HLE. Beyond medical management, a coordinated community and healthcare response is crucial to prevent recurrence and support long-term recovery. Clinicians should screen for substance use at every opportunity and offer compassionate, nonjudgmental counselling.

Expanding access to comprehensive addiction treatment programs, including medication-assisted therapy (MAT), behavioral counselling, and peer support networks, is essential. Collaboration between healthcare providers, addiction specialists, and social workers can help patients overcome barriers to treatment. In addition, public health initiatives aimed at harm reduction-such as increasing availability of naloxone, needle exchange programs, and education about safer use and early warning signs of toxicity-play a critical role in reducing morbidity and mortality associated with heroin use.
